# The complete chloroplast genome sequence of *Taxodium ascendens × T. mucronatum* hybrid (Cupressaceae)

**DOI:** 10.1080/23802359.2021.1899868

**Published:** 2021-03-21

**Authors:** Fan Zhang, Lei Xuan, Yanwei Zhou, Yunlong Yin, Xiaoqing Lu

**Affiliations:** Jiangsu Key Laboratory for the Research and Utilization of Plant Resources, Institute of Botany, Jiangsu Province and Chinese Academy of Sciences, Nanjing, Jiangsu, PR China

**Keywords:** *Taxodium* ‘Zhongshanshan 401’, complete chloroplast genome, phylogenetic analysis

## Abstract

*Taxodium* ‘Zhongshanshan 401’ is an important economic plant with ornamental and ecological values, and has been widely planted in southeastern China. In this study, the complete chloroplast (cp) genome of *T*. ‘Zhongshanshan 401’ was sequenced and illustrated to add the more genetic information. The entire cp genome of *T*. ‘Zhongshanshan 401’ was 132,037 bp in length with 35.3% overall GC content. The cp genome contained 120 genes, including 83 protein-coding genes, 33 *tRNA* genes, and four *rRNA* genes. Fifteen genes contain two exons and two contains three exons. Phylogenetic analysis based on whole cp genome sequences showed that *T*. ‘Zhongshanshan 401’ was more closely related to *T*. mucronatum.

The genus *Taxodium* (L.) Rich., belongs to the family Cupressaceae, is historically recognized as containing three species: *T. distichum* (L.) Rich. (baldcypress), *T. mucronatum* Gordon (Montezuma cypress), and *T. ascendens* (Nutt.) Croom (pondcypress) (Denny and Arnold 2007). *Taxodium* ‘Zhongshanshan’ are selected from the controlled *Taxodium* hybridization, which combine the best characteristics of superior parents (Creech et al. 2011; Qi et al. 2014). Of them, *T.* ‘Zhongshanshan 401’ (*T. ascendens* × *T. mucronatum*) was selected for growth rate and ornamental value, and has been widely used in coastal floodplains in southeastern China. At present, most of the studies of *Taxodium* focused on cultivation and tolerance of abiotic stress (Zhou et al. 2010; Wang et al. 2017), while the genetic resources for *Taxodium* hybrids are still poorly investigated. In this study, we assembled the complete chloroplast (cp) genome sequence of *T.* ‘Zhongshanshan 401’ with bioinformatics analysis, which would be helpful for future cp genetic analysis and molecular breeding studies of *Taxodium* genus.

Fresh leaves of *T.* ‘Zhongshanshan 401’ were collected from Nanjing Botanical Garden, Mem. Sun Yat-sen (118°49′55″E, 32°3′32″N), Nanjing, China. The voucher specimen (NO.NBGJIB-*Taxodium*-0401) was deposited in the Institute of Botany, Jiangsu Province, and Chinese Academy of Science. Total genomic DNA was extracted using the GMS16011.2.1 Kit (Genmed Scientifics Inc., Wilmington, DE). The Illumina NovaSeq platform (Illumina, San Diego, CA) was used to sequence the complete cp genome of *T.* ‘Zhongshanshan 401 with paired-end reads of 150 bp. Whereafter, the NOVOPlasty (Dierckxsens et al. 2017) and GeSeq software (Tillich et al. 2017) were used to assembly and annotation the complete cp genome, respectively. The annotated cp genome has been submitted to GenBank with the accession number MW307789.

The cp genome of *T.* ‘Zhongshanshan 401’ is 132,037 bp in length as a circular form. The structure and gene order of *T.* ‘Zhongshanshan 401’ cp genome are similar to other *Taxodium* species (Duan et al. 2020), which did not have the typical quadripartite structure. There are 120 unique genes in the complete cp genome of *T.* ‘Zhongshanshan 401’, including 33 transfer *RNA* genes, four ribosomal *RNA* genes, and 83 protein-coding genes. A total of 17 genes contained two (15 genes) or three (*ycf3* and *rps12*) exons. The nucleotide composition of cp genome was biased toward A and T, with 64.7% of A + T content (A 32.9%, T 31.9%, C 17.9%, and G 17.4%).

To analyze the phylogenetic status of *T.* ‘Zhongshanshan 401’, 19 other cp genome sequences were obtained from the Genebank database. MAFFT (Katoh et al. 2019) was used to align the 61 protein-coding genes, and neighbor-joining method (bootstrap repeat is 1000) were used for constructing phylogenetic trees using Geneious Tree Builder (http://www.geneious.com). The phylogenetic analysis showed that *T. * ‘Zhongshanshan 401’ was more closely related to *T. mucronatum* species (Figure 1). The cp genome sequence of *T.* ‘Zhongshanshan 401’ in this study will supply useful genetic information for molecular markers and molecular breeding for this valuable tree species.

**Figure 1.**
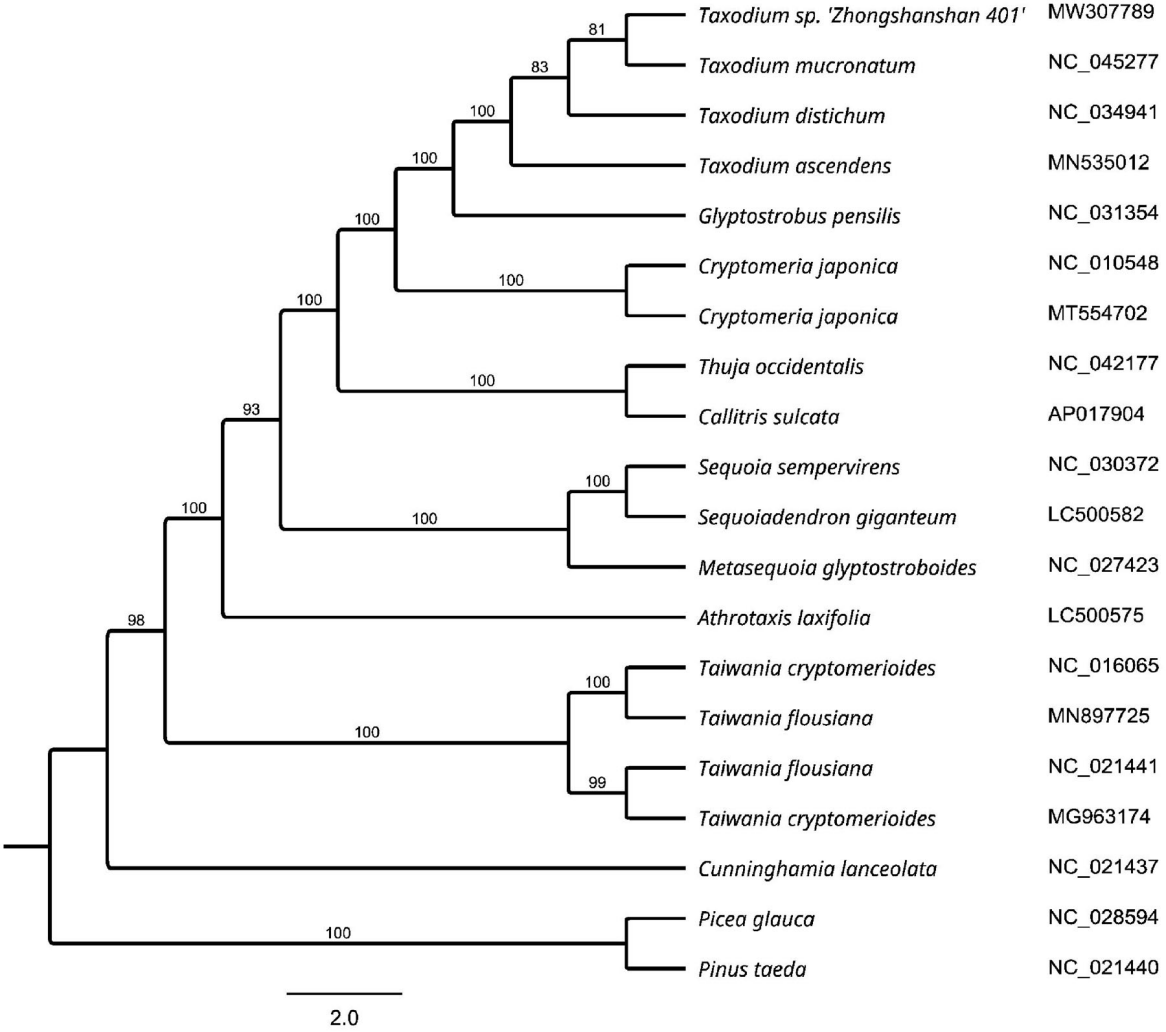
NJ phylogenetic tree of *T*. ‘Zhongshanshan 401’ with 19 related species based on 61 protein-coding genes. Numbers on the nodes are bootstrap values from 1000 replicates. The chloroplast sequence of *Pinus taeda* and *Picea glauca* were set as outgroups.

## Data Availability

The complete cp genome sequence of *T.* ‘Zhongshanshan 401’ is deposited in the GenBank of NCBI (https://www.ncbi.nlm.nih.gov) database under the accession number MW307789. The associated Bioproject numbers is SRR13347062.
